# Supplementary figures and images for: Valley Fever: Environmental Risk Factors and Exposure Pathways Deduced from Field Measurements in California
Source: Int J Environ Res Public Health. 2020 Jul 22;17(15):5285. doi: 10.3390/ijerph17155285 (PMC7432779; doi:10.3390/ijerph17155285)

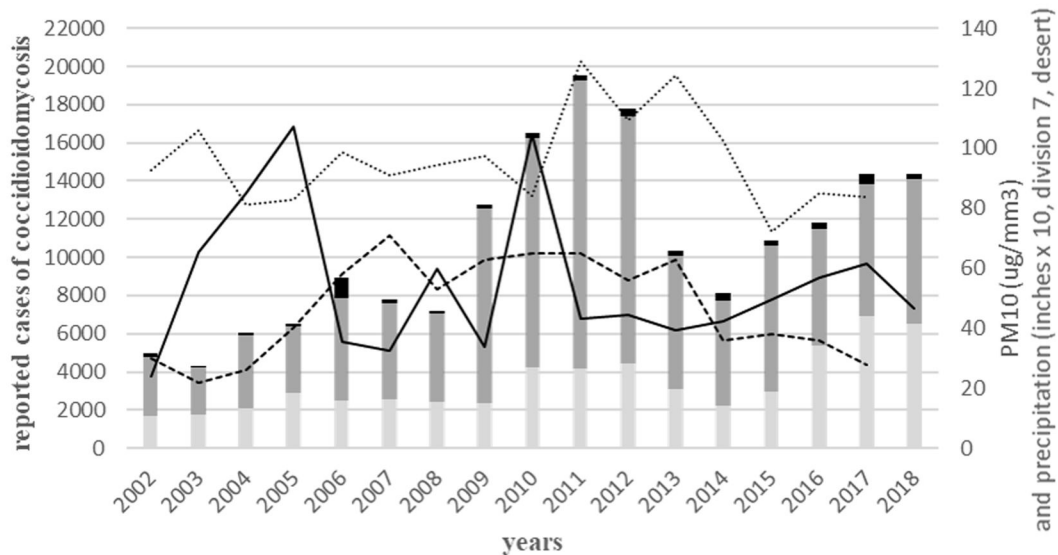

Supplement: Supplementary file 1 [file ijerph-17-05285-s001.pdf]
